# The effects of old and recent migration waves in the distribution of HBB*S globin gene haplotypes

**DOI:** 10.1590/1678-4685-GMB-2016-0032

**Published:** 2016-10-03

**Authors:** Juliana D. Lindenau, Sandrine C. Wagner, Simone M. de Castro, Mara H. Hutz

**Affiliations:** 1 Departamento de Genética, Universidade Federal do Rio Grande do Sul, Porto Alegre, RS, Brazil.; 2 Universidade Federal de Ciencias da Saude, Porto Alegre, RS, Brazil.; 3 Faculdade de Farmácia, Universidade Federal do Rio Grande do Sul, Porto Alegre, RS, Brazil.

**Keywords:** β^S^ globin haplotypes, sickle cell disease, Hemoglobin S, migration

## Abstract

Sickle cell hemoglobin is the result of a mutation at the sixth amino acid position of the beta (β) globin chain. The HBB*S gene is in linkage disequilibrium with five main haplotypes in the β-globin-like gene cluster named according to their ethnic and geographic origins: Bantu (CAR), Benin (BEN), Senegal (SEN), Cameroon (CAM) and Arabian-Indian (ARAB). These haplotypes demonstrated that the sickle cell mutation arose independently at least five times in human history. The distribution of β^S^ haplotypes among Brazilian populations showed a predominance of the CAR haplotype. American populations were clustered in two groups defined by CAR or BEN haplotype frequencies. This scenario is compatible with historical records about the slave trade in the Americas. When all world populations where the sickle cell gene occurs were analyzed, three clusters were disclosed based on CAR, BEN or ARAB haplotype predominance. These patterns may change in the next decades due to recent migrations waves. Since these haplotypes show different clinical characteristics, these recent migrations events raise the necessity to develop optimized public health programs for sickle cell disease screening and management.

## Introduction

Sickle cell hemoglobin is the result of a single nucleotide mutation (GAG→GTG) at the sixth amino acid position of the beta (β) globin gene (HBB). Sickle cell anemia (SCA) is caused by HBB*S homozygosity. This gene has a worldwide distribution (Piel *et al.*, 2010). The disease is a severe chronic hemolytic anemia, but its clinical course is highly variable. Although not completely understood, many factors have been suggested to be modulators of this variability, such as coinheritance with Hb C, α and β thalassemias, as well as high fetal hemoglobin (HB F) levels (Higgs *et al.*, 1982; Frenette and Atweh, 2007).

The HBB*S gene is in linkage disequilibrium with five main haplotypes defined by single nucleotide polymorphisms (SNPs) in the β-globin-like gene cluster. These haplotypes are named according to their ethnic and geographic origins: Bantu (CAR, originated in South-Central and East Africa), Benin (BEN, in Midwest Africa), Senegal (SEN, in Atlantic West Africa),Cameroon (CAM, along the west coast of Africa), and Arabian-Indian (ARAB, from the Indian subcontinent and the eastern Arabian peninsula). Based on this haplotype distribution it has been demonstrated that the HBB*S mutation arose at least five times in human history (Pagnier *et al.*, 1984; Kulozik *et al.*, 1986; Lapouméroulie *et al.*, 1992). Moreover these haplotypes have also been investigated in association with clinical features of the disease in order to disclose if some characteristics associated with disease severity such as HB F levels were also associated with a specific haplotype (Steinberg, 2009). It is essential to know about the old and recent dispersions of these haplotypes considering their clinical heterogeneities and their implications to public health programs for sickle cell disease screening and management.

HBB*S haplotypes have been studied in different Brazilian populations (Table 1), as tools to clarify population origins, since the sickle cell mutation is absent among Native Americans and it was introduced into the American continent basically by gene flow from Africa during the slave trade from the 16th to the 19th century (Zago *et al.*, 1995; Salzano and Bortolini, 2002). In this study, we compared the HBB*S haplotypes frequencies in sickle cell disease patients from several world populations, in order to disclose the effects of old and recent wave migrations in the distribution of HBB*S haplotypes.

**Table 1:** Frequency (%) of HBB*S haplotypes in Brazilian populations.

Population	Haplotypes							Reference
	N	CAR	BEN	SEN	CAM	ARAB	Atypical	
Belém (PA)	60	66.7	30.0	3.3	–	–	–	Pante-de-Sousa *et al*., 1998
Belém (PA)	260	66.0	21.8	10.9	1.3	–	–	Cardoso and Guerreiro, 2006
Ceará (CE)	44	31.8	43.2	2.3	–	–	22.7	Galiza Neto *et al.*, 2005
Ceará (CE)	68	66.2	22.1	–	–	–	11.8	Silva *et al.*, 2009
Rio Grande do Norte (RN)	94	75.5	12.8	–	6.4	–	5.3	Cabral *et al*., 2011
Pernambuco (PE)	127	81.1	14.2	–	0.8	–	3.9	Bezerra *et al*., 2007
Salvador (BA)	72	48.6	51.4	–	–	–	–	Costa *et al*., 1984
Salvador (BA)	160	48.1	45.6	0.6	–	–	5.6	Gonçalves *et al*., 2003
Salvador (BA)	250	41.6	55.2	0.4	1.2	0.4	1.2	Adorno *et al*., 2008
Rio de Janeiro (RJ)	148	54.1	44.6	1.4	–	–	–	Fleury, 2007
São Paulo (SP)	74	64.9	14.9	1.4	–	–	18.9	Zago *et al*., 1992
São Paulo (SP)	148	62.2	33.8	–	–	–	4.1	Gonçalves *et al*., 1994
São Paulo (SP)	74	60.8	36.5	–	–	–	2.7	Costa *et al*., 1984
Rio Grande do Sul (RS)	220	67.3	25.0	0.5	0.9	–	6.4	Present study

N: number of chromosomes;

## Material and Methods

A systematic review was performed to find studies that describe sickle cell haplotypes in different world populations. When more than one study from the same population was available, mean haplotype frequencies were calculated. A Wright's F_ST_ (Weir and Hill, 2002) analysis was performed using ARLEQUIN 3.0 (Excoffier *et al.*, 2005) to determine the differentiation among populations based on haplotype frequencies. Principal component analysis (PCA) was performed to summarize the distribution of populations based on the pairwise F_ST_ using SPSS v.18 software.

This study also included information about 110 non-consanguineous SCD patients from Rio Grande do Sul, southern region of Brazil, screened using isoeletric focusing (IEF) and/or cation exchange high performance liquid chromatography (HPLC) and confirmed by a PCR-RFLP approach with *Dde*I enzyme (Wagner *et al.*, 2010). All patients were ascertained by the Neonatal Screening Reference Service or health care centers. The Ethics Committee of the Federal University of Rio Grande do Sul approved the study protocol.

Genomic DNA was isolated from peripheral blood samples using a salting out procedure (Lahiri and Nurnberger Jr, 1991). Haplotype analysis was performed by PCR-RFLP for the following polymorphic sites in the β globin gene cluster: *Hind*III-Gγ,*Hind*III-Aγ, *Hinc*II-ψβ, *Hinc*II, 3'ψβ, *Hinf*I- 5'β as previously described (Sutton *et al.*, 1989). Haplotypes were inferred using the Multiple Locus Haplotype Analysis program (Long, 1999).

## Results and Discussion

HBB*S haplotypes identified in several Brazilian populations are shown in Table 1. The CAR haplotype was the most frequent one, followed by the BEN haplotype. These results are in accordance with historical reports on slave traffic to Brazil. It is estimated that during the period between 1701 and 1816, 68% of the imported slaves came from Angola and the remainder from the Benin region. From 1843 to 1871, 90% of slaves came from Congo, Angola and Mozambique (Curtain, 1969). The SEN haplotype has its higher frequency in Brazil in Belem, in the northern region (Cardoso and Guerreiro, 2006). This is in accordance on what was expected based on the slave trade historical data of Atlantic West African populations to northern Brazil (10%), considering the high frequency of this haplotype in Senegal (Currat *et al.*, 2002). The CAM haplotype was always in lower frequencies, with 0,9% in Rio Grande do Sul and 0.9-1.3% in other Brazilian regions, probably due to domestic slave trade and later internal migrations from regions supplied with slaves from Central West Africa, where this haplotype has been found (Oner *et al.*, 1992). These results confirmed the diversity of the African influence in Brazilian regions.

**Figure 1:**
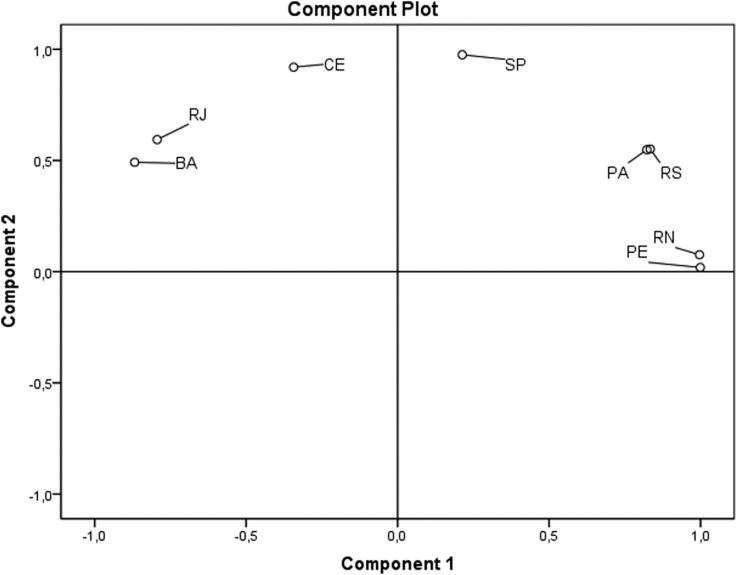
PCA based on F_ST_ distances calculated using haplotype frequencies showing clustering patterns for different Brazilian populations according to HBB*S haplotypes.

PCA (Figure 1) demonstrated that two components explained 98.9% of the variance observed among Brazilians. The first component showed a group composed by Rio Grande do Sul (RS), Pará (PA), Pernambuco (PE), São Paulo (SP) and Rio Grande do Norte (RN) populations, where the CAR haplotype has a high frequency (from 66 to 81%). The other group was composed by Rio de Janeiro (RJ), Bahia (BA) and Ceará (CE) populations, where the CAR and BEN haplotypes have similar frequencies.

**Figure 2:**
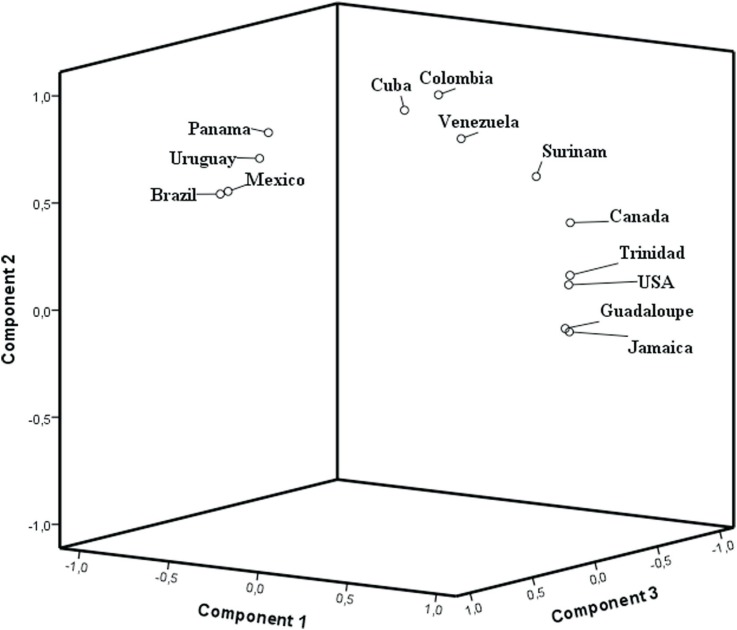
PCA based on F_ST_ distances calculated using haplotype frequencies showing clustering patterns for different American populations according to HBB*S haplotypes.

The Brazilian populations were then compared to other American populations. The PCA (Figure 2) showed the American populations distributed in different clusters. In this analysis, three groups explained 98.9% of the variance observed. Populations with higher frequencies of CAR are clustered together (Uruguay, Brazil, Panama and Mexico), whereas populations with higher BEN frequencies formed another cluster (USA, Canada, Trinidad, Guadeloupe and Jamaica). The other populations present similar BEN and CAR haplotype frequencies and formed a third cluster comprising Venezuela, Suriname, Colombia and Cuba. This cluster pattern appears to reflect geographical data, since a North, Central and South America separation can be observed, except for Mexico. This distribution could also be explained by historical reports of colonial power in these countries: Spain, France and Great Britain (Curtain, 1969). The British and French bought slaves from Midwestern African regions, where the BEN haplotype was prevalent, while slaves imported by the Spanish and Portuguese colonizers were mainly from Atlantic Central Africa, where CAR haplotype was the most prevalent.

**Figure 3:**
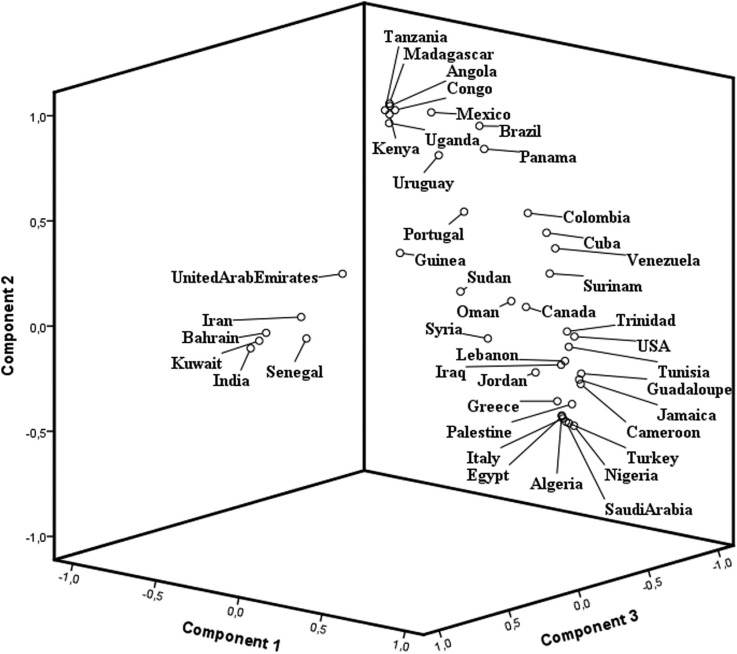
PCA based on F_ST_ distances calculated using haplotype frequencies showing clustering patterns for different world populations according to HBB*S haplotypes.

Table 2 and the PCA of world populations (Figure 3) showed the distribution expected according to the haplotypes' distribution and origin. Three different components could be observed with ARAB, CAR or BEN haplotype predominance. The first group was composed by Kuwait, Bahrain, Iran, India, United Arab Emirates and Senegal. All of them have a predominance of the Arabian-Indian (ARAB) haplotype, except Senegal. The second group was composed by Madagascar, Mexico, Angola, Tanzania, Kenya, Congo, Uganda, Brazil, Uruguay and Panama. All of them have a predominance of the Bantu (CAR) haplotype. The third group was composed by USA, Jordan, Tunisia, Guadeloupe, Canada, Jamaica, Suriname, Greece, Cameroon, Oman, Palestine, Algeria, Venezuela, Egypt, Syria, Cuba, Saudi Arabia, Turkey, Nigeria, Colombia, Sudan, Portugal and Italy. These populations have a predominance of the Benin (BEN) haplotype. The trade slave to the Americas and migration routes to the Mediterranean areas and the Middle East from West Africa determines the BEN haplotype predominance in these regions. Finally, the ARAB haplotype predominated in areas where it was originally derived.

**Table 2:** Frequency (%) of HBB*S haplotypes in different world populations.

Continents	Population	N	Haplotypes						Reference
			CAR	BEN	SEN	CAM	ARAB	Atypical	
Africa	Algeria	20	–	100.0	–	–	–	–	Pagnier *et al*., 1984
	Angola	44	95.5	4.5	–	–	–	–	Lavinha *et al*., 1992
	Cameroon	1082	0.5	73.8	0.2	19.1	0.3	6.1	Bitoungui *et al*., 2015
	Congo	232	90.9	9.1	–	–	–	–	Mouélé *et al*., 1999
	Egypt	28	–	100.0	–	–	–	–	El-Hazmi *et al*., 1999
	Guinea	40	22.5	–	–	77.5	–	–	Sow *et al*., 1995
	Kenya	111	98.2	1.8	–	–	–	–	Ojwang *et al*., 1987
	Madagascar	35	91.4	–	2.9	–	–	5.7	Hewitt *et al*., 1996
	Mauritania	90	4.4	8.9	77.8	–	5.6	3.3	Veten *et al*., 2012
	Nigeria	669	0.9	93.3	–	3.4	–	2.4	Adekile *et al*., 1992
	Senegal	90	–	–	100.0	–	–	–	Currat *et al*., 2002
	Sudan	143	2.8	29.4	18.2	35.0	–	14.7	Elderdery *et al*., 2012
	Tanzania	41	100.0	–	–	–	–	–	Oner *et al*., 1992
	Tunisia	332	2.7	60.5	–	–	–	36.7	Moumni *et al*., 2011
	Uganda	208	99.5	–	0.5	–	–	–	Mpalampa *et al*., 2012
America	Brazil	1176	65.0	31.5	3.0	0.5	–	–	*
	Canada	61	11.5	49.2	13.1	13.1	–	13.1	Oner *et al*., 1992
	Colombia	229	29.7	33.2	4.4	4.4	0.4	27.9	Fong *et al.*, 2013
	Cuba	198	40.9	51.0	8.1	–	–	–	Muniz *et al*., 1995
	Guadeloupe	830	11.1	74.9	6.1	2.3	0.7	5.1	Kéclard *et al*., 1997
	Jamaica	446	8.3	76.0	5.2	–	–	10.5	Mpalampa *et al*., 2012
	Mexico	33	78.8	18.2	–	–	–	3.0	Magaña *et al*., 2002
	Panama	200	51.0	30.0	8.5	4.0	1.0	5.5	Rusanova *et al*., 2011
	Surinam	77	29.9	53.2	2.6	2.6	–	11.7	Oner *et al*., 1992
	Trinidad	283	17.3	61.8	8.5	3.5	3.2	5.6	Jones-Lecointe *et al*., 2008
	USA	806	16.0	62.4	9.4	4.7	1.5	6.0	Crawford *et al*., 2002
	Uruguay	10	60.0	20.0	–	–	–	20.0	Luz *et al*., 2006
	Venezuela	359	36.4	51.5	10.6	1.5	–	–	**
Asia	Bahrain	37	5.4	2.7	–	–	89.2	2.7	Al-Arrayed and Haltes, 1995
	India	140	–	–	–	–	91.4	8.6	Mukherjee *et al*., 2004
	Iraq	128	7.8	69.5	–	–	12.5	10.2	Al-Allawi *et al*., 2012
	Iran	162	3.1	11.7	3.7	2.5	53.7	25.3	Rahimi *et al*., 2003
	Jordan	20	–	80.0	–	–	20.0	–	El-Hazmi *et al*., 1999
	Kuwait	125	5.6	11.2	–	–	80.8	2.4	Adekile and Haider, 1996
	Lebanon	100	15.0	73.0	–	–	10.0	2.0	Inati *et al*., 2003
	Oman	117	21.4	52.1	–	–	26.5	–	Daar *et al*., 2000
	Palestine	118	5.1	88.1	–	–	–	6.8	Samarah *et al*., 2009
	Saudi-Arabia	124	–	98.4	–	–	1.6	–	El-Hazmi *et al*., 1999
	Syria	18	–	66.7	–	–	33.3	–	El-Hazmi *et al*., 1999
	United Arab Emirates	94	25.5	22.3	–	–	52.1	–	El-Kalla and Baysal, 1998
Europe	Greece	14	–	92.9	7.1	–	–	–	Oner *et al*., 1992
	Italy	64	–	100.0	–	–	–	–	Schilirò *et al*., 1992
	Portugal	33	42.4	36.4	21.2	–	–	–	Lavinha *et al*., 1992
	Turkey	214	–	96.3	–	–	0.5	3.3	Oner *et al*., 1992

N: number of chromosomes;

* mean frequency for Brazilian populations showed in Table 1;

** mean frequency for Arends *et al*., 2000; Moreno *et al.*, 2002.

**Figure 4:**
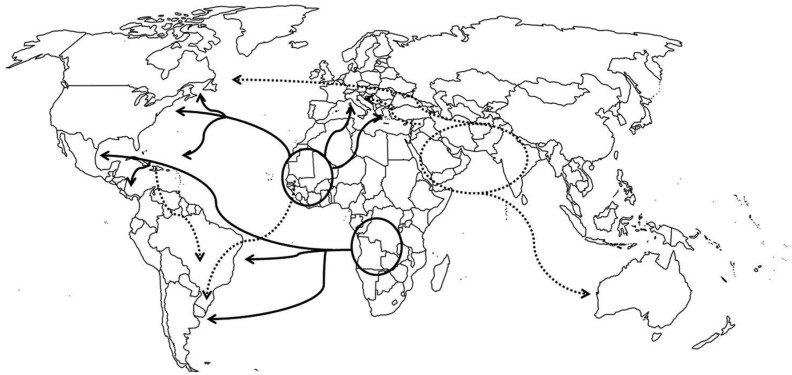
World map showing the main migrations concerning HBB*S dispersion. The full lines represent the old migrations, while the dotted lines represent recent migrations.

This clear pattern of origin and dispersal of HBB*S haplotypes can suffer radical changes in the next decades due to global migrations. At present, the mobility of humans has reached unimaginable levels. This mobility can affect the epidemiology of several diseases, with an increase in the risk of a local disease spreading globally and the introduction of deleterious alleles into populations in which they were previously absent. Information about the number of international migrants in the last decades showed a noticeable difference between migrants with and without HB S. Whereas the number of migrants without HB S increased from 92.6 million in 1960 to 165.2 million in 2000, the number of migrants with this hemoglobin increased faster (from 1.6 million in 1960 to 3.6 million in 2000) (Piel *et al.*, 2014). The estimated number of migrants from African countries, India and Middle East with HB S moving to North America, Western Europe and Australia increased (Piel *et al.*, 2014). An increase in the Arab-Indian haplotype frequency in several countries in the next decades could potentially be expected due migration processes that are occurring from the Middle East to Europe (Figure 4).

A similar process can also be observed in Brazil, where the number of migrants from Bolivia, Haiti and Senegal increased in the last years. The dispersal of these migrants is still uneven, but Bolivians tend to remain in São Paulo state while Senegalese individuals tend to move to Rio Grande do Sul (Figure 4). Therefore, an increase in the contribution of the Senegal haplotype is expected in southern Brazil, reflecting this new migration process. No studies about HBB*S haplotypes in Haiti population are available. This country does not have any national newborn screening program to measure the prevalence of SCD. Nevertheless, a study with infants born in Port-au-Prince showed that the prevalence of SCD in Haitian newborns appears to be more than twice higher than that found among African Americans in the United States (Rotz *et al.*, 2013). This study showed a prevalence of the SCD genotypes Hb SS and HbSC of 1:173 newborns. The authors discuss the importance to consider these results carefully, since many children are born outside hospitals in Haiti, and therefore this prevalence may probably be an underestimate (Rotz *et al.*, 2013). Since Haiti was colonized by French the most probable frequent haplotype would be BEN, as observed in Guadeloupe (Kéclard *et al.*, 1997). Considering this information, independent from the HBB*S haplotype that predominates in these migrants, an increase in HB S prevalence in Brazil is expected in the next years. It is important to consider that the effect of migration cannot be assessed only by the number of migrants, but also by their behavior and habits. In this context, it is essential to consider thata higher intermarriage rate is likely among migrants from the same group, leading to an increase in sickle cell disease prevalence. Some religious or cultural beliefs could be also a factor complicating an effective genetic counseling. The public health system agents should be prepared to address these problems in the best way possible.

Several chromosomes were identified as atypical (chromosomes with less common haplotypes) in all populations. Some of these atypical haplotypes were previously studied and diverse genetic mechanisms were inferred as involved in their origin, such as recombination, point substitutions, or nonreciprocal sequence transfer (conversion) in the pre-existing common haplotypes instead of recurrent *de novo* HBB*S mutations (Zago *et al.*, 2000). Subsequently, it was demonstrated that these events can be observed in typical HBB*S haplotypes in a way similar to those that generate atypical haplotypes (Zago *et al.*, 2001). An extended haplotype within the HBB gene cluster is composed by three elements: a four repeats sequences configuration (AT)*x*N12(AT)*y* motif within the 5' *HS2* region of β*-LCR* site, (TG)*n* (CG)n motif within IVSII region of fetal globin gene (^G^γ and ^A^γ), and (AT)*x*T*y* motif within 5' region of β*-globin* gene region. Molecular investigations of this extended haplotype confirmed that the atypical haplotypes are obtained through recombination among the classical SNPs in the β-globin-like gene cluster and these sites in the extended haplotype region (Moumni *et al.*, 2014).

In addition to population origin effects, these waves of migration can have important effects on public health. It was well established that there is a substantial phenotypic heterogeneity among patients with sickle cell anemia. In general, carriers of the CAR haplotype have the most severe clinical course, while carriers of the Senegal or Arab-Indian haplotypes have the best clinical course. Carriers of the BEN haplotype are intermediate in this respect. As HBB*S presence alone cannot explain this heterogeneity among patients, environmental influences and variations in others genes are likely to modulate the sickle cell anemia phenotype. The main pathophysiological factor determining disease severity is the Hb F concentration, leading to a reduced severity in patients with higher concentrations of this hemoglobin. In addition to Hb F concentration, α-thalassemia can also affect the disease phenotype because both decrease Hb S polymerization. Several genetic and epigenetic factors modulate Hb F levels, such as the locus control region (LCR), the Hb F-related quantitative trait locus (QTL) and secretion-associated and RAS-related gene (*SAR1A*). In addition, several SNPs in candidate genes have been associated with subphenotypes of sickle cell anemia. For example, nonhemorrhagic stroke has been associated with variation in *VCAM1, TNFA, ADRB2, IL4R, LDLR, HLA, ANXA2, SELP* and *TGF-*β*/BMP* genes (a complete review about this topic could be found in Steinberg, 2009).

Considering the possible increase in Hb S frequency in Brazil due the recent wave migrations, it should be important to consider a more appropriate public health policy, including screening, adequate care and counseling, not only to Brazilians but also to migrants. Sometimes it could be difficult for migrants to have full access to public health services due to linguistic, cultural, religious, and social barriers but the government's role is to provide the best opportunities to everyone.
